# Pulmonary thrombectomy and extracorporeal membrane oxygenation: a case study

**DOI:** 10.1051/ject/2025024

**Published:** 2025-09-15

**Authors:** Mariah DeRenzo

**Affiliations:** 1 Emory University Perfusion Program, Emory University Atlanta GA 30322 USA

**Keywords:** Cardiopulmonary bypass (CPB), Pulmonary thrombectomy (PTE), Extracorporeal membrane oxygenation (ECMO)

## Abstract

A 71-year-old male with a history of chronic thromboembolic pulmonary hypertension scheduled for an elective pulmonary thrombectomy was removed from the surgical list in 2019 for unknown reasons. Four years later, a different surgeon elected to perform the surgery with cardiopulmonary bypass support. Following surgery, the patient was placed on extracorporeal membrane oxygenation and ultimately died. This case report highlights the surgical and perfusion techniques, as well as the rare events that occurred during his care.

## Introduction

Pulmonary thrombectomy (PTE) or pulmonary endarterectomy is a surgical procedure used to remove blood clots or fibrous tissue from the pulmonary vasculature. Chronic thromboembolic pulmonary hypertension (CTEPH) is the most common cause of precapillary pulmonary hypertension. CTEPH is characterized by a mean pulmonary pressure greater than 25 mmHg at rest and a pulmonary wedge pressure above 15 mmHg [1]. A patient with a mean pulmonary artery pressure above 50 mmHg has a three-year mortality rate of 90% [2]. Currently, PTE is the only potentially curative measure for the treatment of CTEPH. PTE procedures are rare as they require a high level of surgical skill. Currently, the University of California, San Diego Hospital (UCSD) is the top center for the procedure and has performed 4800 PTEs since 1987, with a mortality rate of 1% [3]. As of 2018, the national average of PTE procedures was 300 per year [2].

The surgical procedure includes placing the patient on cardiopulmonary bypass support, cooling to profound hypothermia [14–20 °C], followed by circulatory arrest, and excision of the fibrous tissue from each pulmonary artery [2]. The length of these procedures is approximately 4–6 h [4].

The most common causes of death post-operatively are right ventricular failure and reperfusion pulmonary edema [1]. Thus, some patients require initiation of extracorporeal membrane oxygenation (ECMO) as a bridge-to-recovery [1]. However, mortality associated with ECMO in this population is high, with a 30-65% survival rate [1]. Pulmonary hemorrhage is documented in 2% of cases [5]. ECMO is used in rare cases of pulmonary hemorrhage to offload the blood flow to the lungs.

## Case description

A 71-year-old male with a history of CTEPH was diagnosed with a pulmonary embolism in 2019. The patient was recommended to undergo a PTE surgical procedure to alleviate his pulmonary hypertension in 2021. However, for unknown reasons, he was dropped from the surgical list. Results of cardiac catheterization revealed the following: Ejection fraction 65–70%, right atrial 16/13 mmHg (mean 14 mmHg), right ventricular (RV) pressure 76/12 mmHg, pulmonary artery (PA) pressure 76/27 mmHg (mean 43.3 mmHg), pulmonary wedge pressure 24 mmHg, and a cardiac output of 7.38 L/min. His baseline blood pressure was 139/71 mmHg, heart rate of 84 bpm, temperature of 36.6°C, respiratory rate of 18 breaths/min, and SaO_2_ of 85%. The patient was 178 cm tall and weighed 102 kg with a BSA of 2.2 m^2^.

During the pre-incision time-out, cannulation and surgical strategy were discussed. The team agreed on central cannulation with a 22 Fr EOPA arterial cannula, a 28 Fr DLP single-stage for the superior vena cava (SVC), and a 29 Fr 3-stage MC2X™ Three Stage Venous Cannula for femoral cannulation EOPA (Medtronic, USA). The conduct of anesthesia was at the discretion of the attending anesthesiologist using narcotic and inhalation agents. Target mean arterial blood pressure and heart rate were maintained within ±20% of mean baseline values. Hemodynamic control was provided by modification of the concentration of anesthetic agents, intravenous vasoactive drugs, and volume repletion [6].

Cerebral oximetry was monitored with near-infrared spectroscopy (INVOS NIRS, Medtronic, USA). Anticoagulation was monitored using a Medtronic Hepcon Hemostasis Management System PLUS (Medtronic USA), while blood gas analysis was obtained via a Werfren GEM Premier 5000 system (Instrumentation Laboratory, USA). Autologous blood recovery was achieved with a Cell Saver^®^ Elite^®^+ with dual reservoirs (Haemonetics, USA).

The cardiopulmonary bypass circuit consisted of the following: Essenz heart-lung bypass system (Liva Nova, USA), and a custom tubing pump pack (Medtronic, USA). A prime volume of 1400 mL of Plasmalyte-A, 10,000 units of heparin, and 100 mL of 25% albumin. The calculated heparin dose was 38,000 units. Following cannulation for CPB, retrograde autologous priming was performed to reduce the prime to 600 mL. The patient was uneventfully placed on CPB and cooled to a bladder temperature of temperature of 14.5 °C using laminar, non-pulsatile flow with a cardiac index ranging between 2.4–2.6 L/min. Before circulatory arrest and at 30-minute intervals, additional heparin was administered to maintain the ACT levels above 480 s with a heparin concentration above ≥300 μ/kg. Standard protocol for PTE procedures utilizes no cross-clamp or cardioplegia, with arrest being induced by ventricular fibrillation secondary to profound hypothermia. The total bypass time was 492 min, with a total circulatory arrest (CA) time was 109 min. The perfusion strategy for this procedure was closely similar to the method developed by UCSD. SvO_2_ of >90% was achieved before each circulatory arrest to ensure adequate oxygenation to previously ischemic tissue. A total of five CAs were performed for this case in parallel with the number of clots present and the difficulty of access. The procedure was as follows: the perfusionist discontinued CPB (pump off), clamped the arterial line, drained the patient volume into the venous reservoir, and then clamped the venous line. During CA, the surgeon was notified every 5 min with a 20-minute maximum per arrest. During CA, FiO_2_ was maintained at 50%, a sweep of 250 mL/min, and isoflurane at 0.5%. During each 10-minute full flow interval, the FiO_2_ was set to 100%, sweep of 4 L/min, and isoflurane at 1%.

Following the repair of the pulmonary arteries, there was no improvement in pulmonary pressure. This may be attributed to right ventricular dysfunction associated with prior distention and inadequate oxygenation from CTEPH. Following unsuccessful weaning from CPB, V-A ECMO was initiated with a 25 Fr. Medtronic cannula inserted into the right femoral artery and a 22 Fr Opti Site (Edwards, USA) cannula placed in the right internal jugular vein with the following settings: a flow 5 L/min, 4000 rpm, sweep of 5.4 L/min, FiO_2_ of 100%, and SvO_2_ 56.3%.

The blood pressure dropped from a MAP of 70 mmHg to 40 mmHg, consequently, the patient was switched to V-VA, with the addition of a 16 Fr Fem-Flex femoral arterial cannula (Edwards, USA) and an 8 Fr DLP^®^ cannula (Medtronic, USA) in the left superficial femoral artery. The ECMO settings at this time were: a flow of 5.4 L/min at 4000 rpm, sweep of 6 L/min, FiO_2_ of 100%, and SvO_2_ of 56.3%.

The following day, the patient had a chest washout and closure. Five days postoperatively, the patient was transitioned to V-V ECMO, placed on a ventilator at 60%, and the sweep was reduced from 6 L/min to 0.5 L/min with flows being weaned down gradually from 5.4 L/min to 4 L/min. On the 6th postoperative day in the ICU, the patient experienced a pulmonary hemorrhage with a 4 L loss, leading to subsequent clamping of the ventilator tube. Seven days postoperatively, the patient’s blood pressure decreased from 133/89 mmHg to 96/55 mmHg, prompting an increase in flow to 5 L/min and a sweeping increase to 9 L/min. Subsequent labs revealed an elevated plasma-free hemoglobin level of 170 mg/dL, which alerted the ECMO specialist team to prep for an oxygenator changeout in case of oxygenator failure. Eight days postoperatively, continuous renal replacement therapy (CRRT) was added to the circuit through a manifold, and the patient was actively cooled secondary to fever development. Indications for CRRT included a rising potassium of 4.9 mmol/L, bilirubin of 24 mg/dL, creatinine of 5.2 mg/dL, uric acid of 22 mg/dL, BUN of 117 mg/dL, eGFR of 10 mL/min/1.73 m^2^, and a urea nitrogen of 117 mg/dL. Ten days postoperatively, CRRT was discontinued, and the patient was rewarmed to 37 °C. Perioperative blood product usage was as follows: RBCs 10 units, FFP 8 units, platelets 7 units, and cryoprecipitate 25 units. Unfortunately, on the 24th postoperative day, the family withdrew care. Institutional review board approval was not required for this case report.

## Discussion

Although PTE was the indicated treatment, the associated risk factors contributed to postoperative complications. The patient’s previous removal from a surgical list for unknown reasons may have been attributed to the complexity and high acuity of the surgical procedure.

Pre-operatively, the patient’s mean PA pressure was 46 mmHg, while the 3-year mortality rate at 50 mmHg was 90% [2]. It is unknown how long the patient had elevated PA pressures.

The PTE was deemed unsuccessful because the PA pressure was unchanged. Transesophageal echocardiogram (TEE) demonstrated poor right ventricular function. Upon collaboration, the surgical team initiated ECMO as a last resort to alleviate the pressure and workload of the PA and RV, respectively. Unfortunately, neither the PTE surgical procedure nor ECMO helped this patient, and after 24 days on ECMO, the family withdrew support. It is also noted that an elevation in plasma-free hemoglobin alone is not sufficient cause to warrant an oxygenator exchange [7]. This patient may be categorized as part of the 2% mortality and 2% pulmonary hemorrhage populations [5], and this case highlights the importance of patient selection for high-risk procedures.

## Conclusion

CTEPH is a serious condition that has only one known curative measure. PTE is an invasive surgical procedure that removes blood clots and fibrous tissue by hand. It takes a very skilled surgeon to perform this operation, and it is only successfully performed in a few centers. The case described highlights a patient with severe pulmonary hypertension at an elevated risk for the PTE procedure. The surgery itself proceeded uneventfully; however, was unsuccessful and the patient required ECMO. While ECMO has proved beneficial in the past, this patient did not improve, and the family chose to withdraw care. The pulmonary hemorrhage seen in this case was a rare occurrence that complicated the overall outcome.

## Data Availability

All available data are incorporated into the article.

## References

[1] Kelava M, Koprivanac M, Smedira N, et al. Extracorporeal membrane oxygenation in pulmonary endarterectomy patients. J Cardiothorac Vasc Anesth. 2019;33:60–69. 10.1053/j.jvca.2018.06.025. 30145074

[2] Madani MM. Surgical treatment of chronic thromboembolic pulmonary hypertension: pulmonary thromboendarterectomy. Methodist Debakey Cardiovasc J. 2016;12(4):213. https://journal.houstonmethodist.org/article/10.14797/mdcj-12-4-213/. 28289496 10.14797/mdcj-12-4-213 PMC5344471

[3] UC San Diego Health. Pulmonary Thromboendarterectomy (PTE) for CTEPH. 2024. Available from: https://health.ucsd.edu/care/heart-vascular/pulmonary-hypertension/pte/.

[4] Cleveland Clinic. Pulmonary Thromboendarterectomy (PTE). 2022. Available from: https://my.clevelandclinic.org/health/treatments/21024-pulmonary-thromboendarterectomy-surgery.

[5] Roscoe AJ, Hwang NC. Pulmonary thromboendarterectomy and pulmonary haemorrhage. Ann Card Anaesth. 2022;25(2):200–201. Available from: https://www.ncbi.nlm.nih.gov/pmc/articles/PMC9244268/. 35417969 10.4103/aca.aca_247_20 PMC9244268

[6] McNair E, Marcoux JA, Bally Cet al. Bivalirudin as an adjunctive anticoagulant to heparin in the treatment of heparin resistance during cardiopulmonary bypass-assisted cardiac surgery. Perfusion. 2016;31(3):189–199. 10.1177/0267659115583525. 25934498

[7] Butt SP, Razzaq N, Saleem Y, Cook B, Abdulaziz S. Improving ECMO therapy: Monitoring oxygenator functionality and identifying key indicators, factors, and considerations for changeout. J Extra Corpor Technol. 2024;56(1):20–29. https://www.ncbi.nlm.nih.gov/pmc/articles/PMC10941833/. 38488715 10.1051/ject/2023047 PMC10941833

